# Taming the genome: towards better genetic test interpretation

**DOI:** 10.1186/s13073-016-0325-9

**Published:** 2016-06-20

**Authors:** Colleen Caleshu, Euan A. Ashley

**Affiliations:** Stanford Center for Inherited Cardiovascular Disease, 300 Pasteur Drive, Stanford, CA 94305 USA; Department of Pediatrics, Division of Medical Genetics, 300 Pasteur Drive, Stanford, CA 94305 USA; Department of Medicine, Division of Cardiovascular Medicine, 300 Pasteur Drive, Stanford, CA 94305 USA; Department of Genetics, Stanford University School of Medicine, 300 Pasteur Drive, Stanford, CA 94305 USA; Stanford Clinical Genome Service, 300 Pasteur Drive, Stanford, CA 94305 USA

## Abstract

Advances in sequencing technology have taught us much about the human genome, including how difficult it is to interpret rare variation. Improvements in genetic test interpretation are likely to come through data sharing, international collaborative efforts to develop disease–gene specific guidelines, and computational analyses using big data.

Genetic testing is a rapidly growing part of medicine. Insurer UnitedHealth estimates that the genetic testing market will increase fivefold in the next 5 years [1]. The number of clinically available genetic tests for rare Mendelian disorders has been rising rapidly, and as of this writing has surpassed 60,000 [1]. In the realm of preventative medicine, projects like Geisinger’s MyCode and National Institutes of Health (NIH) ClinSeq aim to explore the role of sequencing in predicting disease in healthy individuals. Yet, the benefit patients reap from genetic testing is entirely dependent on our ability to interpret the results.

## Insights from broad sequencing initiatives

Several factors have come together in recent years to make the clinical genetics community re-evaluate our approach to the interpretation of molecular genetic tests. Analysis of the exomes of tens of thousands of individuals in projects like those that make up the Exome Aggregation Consortium (ExAC) have revealed that rare variation is far more abundant in the human genome than previously thought [2]. This calls into question both the field’s prior assumption that most rare variation causes severe Mendelian genetic disease and the specific classification of thousands of variants that was based heavily on our prior assumptions regarding rarity. At the same time, data-sharing efforts revealed that laboratories do not always agree in their interpretation of genetic test results. An analysis of nearly 13,000 variants in ClinVar with classifications by more than one laboratory found that in 17 % of cases the laboratories’ classifications differed [3].

**Fig. 1:**
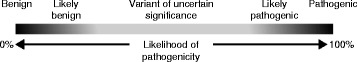
Genetic test interpretation is probabilistic. Variants found through genetic testing are classified as benign, likely benign, uncertain significance, likely pathogenic, or pathogenic. These various classifications fall along a spectrum of likelihood that the variant is disease causing, with benign at one end, pathogenic at the other, and a broad range of uncertainty in the middle. Reproduced with permission from [11]

These data inspire a fair amount of humility. The genome is not easily tamed and as a field we have had to face that we are not as good at interpreting it as we had hoped. Variant interpretation is an inherently challenging endeavor. It is a probabilistic assessment of the likelihood that a given variant is disease-causing (Fig. 1). A myriad of data types inform that assessment and we rarely have all of the data points we would like to have, either because they simply do not exist or because they are not shared publicly.

## New guidelines

It is within this context that the American College of Medical Genetics and Genomics (ACMG) and the Association for Molecular Pathology (AMP) published new recommendations for the interpretation of sequence variants in early 2015 [4]. The new recommendations detailed a scoring system that gave different weights to different types of evidence and leads to a classification of pathogenic, likely pathogenic, uncertain significance, likely benign, or benign. While these categories appear discrete, they really are points on a spectrum of likelihood of pathogenicity (Fig. 1). Clinically, variants deemed pathogenic or likely pathogenic are often used in making diagnoses, assessing risk of disease in healthy relatives, and reproductive genetic testing. These recommendations were intended to provide general guidance with the understanding that both gene-specific knowledge and clinical judgment would be needed for effective implementation.

## Putting the new guidelines into practice

An effort to test the new ACMG–AMP recommendations in practice was published recently in the *American Journal of Human Genetics* [5]. Nine variants were classified by nine laboratories and a further 90 variants were randomly assigned to three of the nine laboratories. The participating laboratories are members of the Clinical Sequencing Exploratory Research Consortium (CSER), an NIH-funded multicenter initiative aimed at generating evidence to guide the application of sequencing to medical care [6]. Using their own internal classification methods, the laboratories agreed on the classification of 34 % of the variants. Interestingly, when the laboratories classified the variants using the new ACMG–AMP criteria their level of agreement did not increase. Through discussion among the laboratories the level of agreement was raised from 34 to 71 %. While 29 % of variants did not reach consensus after discussion, only 5 % differed in a manner likely to affect medical management (i.e., likely pathogenic or pathogenic versus any other classification).

The markedly low level of initial agreement among laboratories using either internal methods or the new ACMG–AMP methods (34 %) is a striking finding. It is not clear what criteria the laboratories used to select these variants. Ascertainment bias could have impacted the observed level of agreement; if the laboratories selected particularly challenging or controversial variants that would have inflated the rate of disagreement. The prior report of 17 % disagreement among submitters in ClinVar may be a better estimate of the overall rate of disagreement in classification between laboratories [3]. Interestingly, we have found a similar rate of disagreement between our clinical team and genetic testing laboratories; in an analysis of 578 variants in our clinical cohort our classification differed from the testing laboratory 20 % of the time (CC, unpublished data).

It is notable and somewhat discouraging that application of the new ACMG–AMP recommendations did not initially increase agreement between laboratories. Interestingly, the authors found that this was partially due to the nuances of how the ACMG–AMP criteria were applied. Some disagreement in classifications occurred because the laboratories used a given rule differently or differed in their decisions about whether a specific rule was met. This suggests that detailed instructions may be needed to ensure consistent and appropriate use of classification rules. Differences in gene–disease specific expertise also played a role in disagreements between laboratories.

## The way forward

While these recent findings may spark some concern regarding the interpretability of sequence-based genetic tests, we believe that there is ample room for hope. Improvements in genetic test interpretation are coming from data sharing, international collaborations to create gene–disease specific guidelines, and insights into pathogenic and benign variation gained from big data. Our understanding of the genome and its variation has improved immensely in recent years and that understanding can be harnessed to improve interpretation of clinical genetic tests.

Both the ACMG–AMP guidelines and the articles by Amendola et al. point out the need for gene and, in some cases, gene–disease specific guidance on elements like allele frequency thresholds for assessing rarity, clinical validity of functional assays, and the role of loss of function variants [4, 5]. Through the ClinGen project, groups comprising international experts on specific diseases are developing just this sort of guidance [3]. For example, the first initiative tackled by ClinGen’s Cardiovascular Domain Working Group has been modification of the original ACMG–AMP recommendations specifically for classification of *MYH7* variants for cardiomyopathy. The majority of the needed modifications were either to remove rules not applicable to *MYH7* or to refine rules to make them more specific to this gene–disease pair. Initial testing of these *MYH7*-cardiomyopathy specific rules shows a high level of concordance [7]. Similar efforts are underway for other disease–gene pairs [4].

Variant interpretation will continue to benefit from increased data sharing, exemplified by projects such as ExAC and databases like ClinVar [2]. Efforts are being made to resolve disagreement among laboratories submitting to ClinVar; in one such effort 15 % of differences could be resolved by sharing internal, unpublished data [8]. While these efforts are both needed and laudable, it is important that we aim not only to agree with one another in our classifications, but also to ensure that our classifications are correct and likely to stand the test of time.

We recently used sequence data on 2913 individuals with hypertrophic cardiomyopathy from the international Sarcomeric Human Cardiomyopathy Registry (SHaRe) and 103,636 individuals not selected for Mendelian disease to dissect which parts of the three-dimensional structure of the myosin heavy chain molecule are intolerant to variation [9]. The findings can help laboratories and clinicians assess the likelihood of pathogenicity of any *MYH7* variant. At the gene level, Walsh et al. recently compared the burden of rare variation in 7855 cardiomyopathy cases and 60,706 ExAC exomes to assess the strength of reported associations between select genes and hereditary cardiomyopathies [10]. Analyses like these are only possible with large-scale international data sharing.

Clinicians also have to be active and critical consumers, carefully reviewing the rationale for the classifications they receive from genetic testing companies. This includes checking ClinVar, reviewing the primary data, and, if possible, making their own assessments of the appropriate classification. We have found this has become common practice among cardiovascular genetics groups like ours, with 81 % of cardiovascular genetic counselors reporting that their clinical team assesses the classification of variants they receive through clinical genetic testing. Periodic re-assessment of classifications is critical as medically impactful classification changes will occur in a subset. Outdated classifications were responsible for nearly a quarter of discordance in ClinVar in one study [8].

## Conclusion

Genetic test interpretation remains challenging, with a significant need for improved methods. Effective variant interpretation guidelines should be gene–disease specific, data driven, and precise in implementation instructions. Sharing of case data by clinical laboratories, researchers, and clinicians alike is critical to optimization of variant classification. Comparison of rare variation in large cohorts of Mendelian disease cases and unselected individuals is providing valuable insight into the location and nature of pathogenic variation, which can in turn inform genetic test interpretation. Combined with our rapidly growing understanding of the genome, these efforts offer hope for improvement in our ability to make meaningful use of genomic variation in medical care.

## Abbreviations

ACMG, American College of Medical Genetics and Genomics; AMP, Association for Molecular Pathology; ExAC, Exome Aggregation Consortium; NIH, National Institutes of Health
